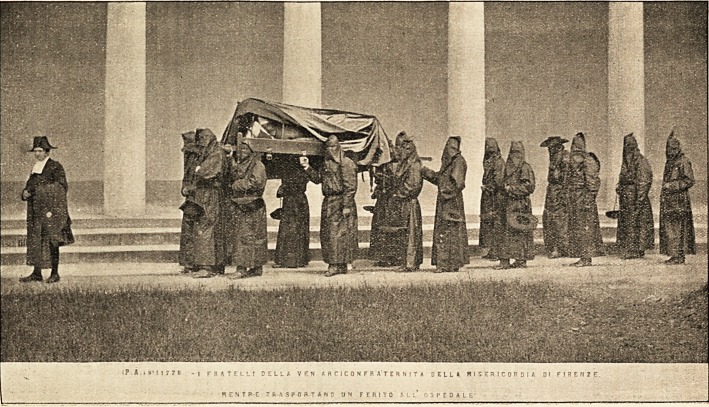# Scraps

**Published:** 1899-03

**Authors:** 


### SCRAPS

PICKED UP BY THE ASSISTANT-EDITOR.

**The Misericordia.**—The Roman correspondent of the *Lancet*, writing in the issue of February 25th, says:—"When the 'Romance of Outdoor Relief' comes to be written it will owe some of its most touching, most picturesque, chapters to the Misericordia—a brotherhood peculiar to Tuscany, seen at its best in Florence, though also admirably *en évidence* at Siena, Pisa, Leghorn, and Lucca, and not unworthily represented in the minor provincial towns, inland or maritime. Ecclesiastical in origin, it was founded in 1244 by Pietro di Luca Borsi and its *personnel* is drawn from every social rank, all and individually bound to serve whenever summoned, without fee or reward. The Grand Duke himself, when presiding at a State banquet in the Pitti Palace, has had to rise and leave his guests when his turn came and to bear a hand with tradesmen, nobles, mechanics, professional men—with the company, in fact, promiscuously improvised to transport to hospital some victim of an accident or to carry a patient from the sick bed to the suburban lodging indicated by the physician. The service is not one of 'unskilled labour.' The members of the brotherhood have all been previously trained to lift the sufferer from the street, to turn the patient in bed, and put him on the 'bara' or stretcher with the minimum of pain or of risk to compromised limbs or organs, and thereafter to bear him through the thoroughfares to his destination with the least possible vibration, friction, or disturbance. As often happens, the particular company told off on sudden duty is composed of men as various in altitude as they are in social position, so that in carrying the 'bara' shoulder high they employ for the first part of the journey those of them who are as nearly as possible of the same height, and when these are tired they lower their burden to the less tall without interruption of movement or alteration of pace and so continue the shifting process till the sufferer is at his journey's end and laid down in bed with scarcely the consciousness of having been transported at all. Few sights or sounds are more impressive in the Florence of to-day than the 'measured march' of the Misericordia through its crowded streets, as robed in black gowns and hooded in black cowls with

(P. A.) N. 11776 — I FRATELLI DELLA VEN. ARCICONFRATERNITA DELLA MISERICORDIA DI FIRENZE MENTRE TRASPORTANO UN FERITO ALL'OSPEDALE

openings for the eyes the brotherhood wends its way with its burden, the bystander lifting his hat sympathetically, the traffic reverently falling aside, and the street noises subdued to a momentary hush in presence of

'The still, sad music of humanity.'

Queen Victoria, it is well known, took profound interest in the Misericordia during her successive sojourns in Florence, and one of its highest office-bearers, the late Cavaliere Cesare Barsi, was deputed by the Arci-Confraternità to visit the Villa Palmieri, there to set forth to Her Majesty its origin and constitution, the nature of its service, the resources at its command, and the more striking incidents in its experience. As I have said, the brotherhood is peculiar to Tuscany, though other cities have their equivalents, each in its own way rendering similar service; few if any of them, however, being able to point to the same antiquity of origin, to the same large resources, or to the same admirable discipline and organisation. I have been led to give this brief notice of the Misericordia from the funeral obsequies just celebrated in Florence of the Provveditore (or general provider and administrator) of the Brotherhood, Signor Giuseppe Bronzuoli—a singularly imposing and picturesque pageant composed of 300 members of the Arci-Confraternità, 50 'capi di guardia' (chiefs of the guard) and 35 priests. The procession included many citizens among whom were not a few surgeons and physicians, those of them who immediately followed the hearse bearing lighted torches."

Canon Wallace, who kindly lent me the photograph from which the picture on the opposite page has been reproduced, sends me the following information, for the authenticity of which he does not vouch, but which he got from apparently good sources:—

In the 13th century, the porters in the piazza of the Duomo, Florence, were a gambling, swearing set. In 1240, Pietro Borsi, one of their number, set on foot a system of fines for bad language, and suggested that the accumulated money should be used for litters and bearers for the sick.

Under the present system, which has been developed out of Borsi's idea, at the sound of the campanile bell, day or night, the members on the roster for that day repair at once to the hall of meeting. The summoning official turns an hour-glass to mark the interval between their summons and arrival. In assigning their task the Captain says: "Brethren, prepare for a work of mercy," then kneeling prays: "Mitte, Domine, nobis caritatem, humilitatem et fortitudinem." They reply: "Ut in hoc opere Te sequamur." He then bids them say a Paternoster for the sufferer. Four take up the litter. The captain leads and four follow. They relieve each other. As they change one set says, "God reward you;" the other replies, "Go ye in peace." Some of the sick nurses are trained specially to move sufferers and patients in accident cases, and are called "Mutanti." They receive no pay, and are allowed to accept only cold water. In sickness a physician is provided for them. They are of "all sorts and conditions of men," and when the official dress is slipped on over their ordinary clothes the rank or occupation of the brother can only be guessed from his boots.

**Clinical Records** (26).—The house-surgeon of a London hospital was attending to the injuries of a woman whose arm had been severely bitten. As he was examining the wound, he said: "What sort of animal bit you? This is too small for a horse's bite, and too large for a dog." "Oh, sir," replied the patient, "it wasn't an animal. It was another lydy."

**Medical Philology** (XXIX.)—The following is to be found in the *Promptorium*: "Deffe, or dulle. *Obtusus, agrestis.*" Mr. Way's note says: "Jamieson observes that deaf signifies properly stupid, and the term is transferred in a more limited sense to the ear. It is also applied to that which has lost its germinating power; thus in the North, as in Devonshire, a rotten nut is called deaf, and barren corn is called deaf corn, an expression literally Ang.-Saxon. An unproductive soil is likewise termed deaf. The plant lamium, or archangel, known by the common name, dead or blind nettle, in the *Promptorium*, has the epithet DEFFE, evidently because it does not possess the stinging property of the true nettle."

Jamieson's statement is not quite accurate. Although the word was used with the general meaning to which he refers, it was also employed in its ordinary restricted sense from very early times. The *New English Dictionary* gives 9th and 13th century instances; and it will be remembered that the Wife of Bath, described by Chaucer, long before the compilation of the *Promptorium*, was "som-del deef, and that was scathe."

The *Promptorium* in another place gives *surdus* as an equivalent of "deffe."

Readers who may be interested in the forms which are figurative or now dialectal will find several quotations in the *New English Dictionary*.

**La Médecine en quatrains.**—Quelqu'un pensa, dit-on, à mettre l'histoire de France en madrigaux. Ce n'était pas une tâche plus extraordinaire que de découper la médecine en quatrains, et plus d'un s'y est essayé.

Il y a les *Aphorismes d'Hippocrate*, mis en vers françois, par le sieur de Launay, chirurgien; Paris, 1642. Il y a les *Quatrains anatomiques des os et des muscles du corps humain*, par le sieur Claude Binet; Lyon, 1664.

La chimie elle-même s'est, parfois, ingénieusement et agréablement alliée à la poésie. En doutez-vous ? Lisez ceci :

Voulez-vous fair' de l'hydrogène ?
Prenez un tub' de porcelaine ;
Mettez-y du fer et de l'eau :
Placez le tout sur un fourneau,
En vapeur l'eau décomposée
Est promptement analysée :
L'oxygène s'unit au fer,
Et l'hydrogèn' s'en va dans l'air.

De la musique d'Hervé là-dessus et ce serait charmant !—*La Revue médicale*.

**Hebrew Therapeutics.**—From a periodical called *The Old Paths*, in which the Rev. Alexander McCaul, D.D., compared "the Principles and Doctrines of Modern Judaism with the Religion of Moses and the Prophets," I extract from the number for July 1st, 1836, the following:—

"Wisdom is a test of true religion, and folly of a false one. Let us then apply this test to the religion of the oral law. Does it commend itself to the understanding by its wisdom, and the wisdom of its teachers ? It is true, that it speaks well of itself, and calls all its doctors 'wise men'; but the histories which the Talmud gives of the Rabbinical practice with regard to charms lead to the inevitable conclusion that wisdom is not one of the characteristics of the oral law. Take for example the following: 'For a bleeding at the nose, let a man be brought who is a priest, and whose name is Levi, and let him write the word Levi backwards. If this cannot be done, get a layman, and let him write the following words backwards—"Ana pipi Shila bar sumki," or let him write these words—"Taam dli bemi keseph, taam li bemi paggan." Or let him take a root of grass, and the cord of an old bed, and paper and saffron, and the red part of the inside of a palm-tree, and let him burn them together, and let him take some wool, and twist two threads, and let him dip them in vinegar, and then roll them in the ashes, and put them into his nose. Or let him look out for a small stream of water that flows from east to west, and let him go and stand with one leg on each side of it, and let him take with his right hand some mud from under his left foot, and with his left hand from under his right foot, and let him twist two threads of wool, and dip them in the mud, and put them into his nostrils. Or let him be placed under a spout, and let water be brought and poured upon him, and let them say, 'As this water ceases to flow, so let the blood of M., the son of the woman N., also cease."' (Gittin, fol. 69, col. 1.) Now we ask any Jew of common sense, whether this passage savours most of wisdom or folly ? Vinegar and water may be very useful in such a case, or even mud, if used in sufficient quantity, might stop up the nose, and therefore stop the bleeding too, but what manner of benefit can proceed from the word Levi written backwards, or from the words which Rashi pronounced to be magical ? Why is the mud of water flowing from east to west more efficacious, and why is it to be taken with the right hand from under the left foot, and with the left hand from under the right foot ? Plainly because the authors of this passage thought there was some charm or magic power, and their minds were so overpowered by superstition as to lead them to disregard the plain words of Moses forbidding all magic. It cannot be pretended that this is a rare case, the Talmud abounds in such remedies, all equally wise."

## Figures and Tables

**Figure f1:**